# Effects of *in Utero* Exposure of C57BL/6J Mice to 2,3,7,8-Tetrachlorodibenzo-*p*-dioxin on Epidermal Permeability Barrier Development and Function

**DOI:** 10.1289/ehp.1308045

**Published:** 2014-06-06

**Authors:** Clarisse S. Muenyi, Sandra Leon Carrion, Lynn A. Jones, Lawrence H. Kennedy, Andrzej T. Slominski, Carrie H. Sutter, Thomas R. Sutter

**Affiliations:** 1 Department of Biological Sciences, and; 2 Department of Chemistry, University of Memphis, Memphis, Tennessee, USA; 3 Department of Pathology, University of Tennessee Health Science Center, Memphis, Tennessee, USA

## Abstract

Background: Development of the epidermal permeability barrier (EPB) is essential for neonatal life. Defects in this barrier are found in many skin diseases such as atopic dermatitis.

Objective: We investigated the effects of 2,3,7,8-tetrachlorodibenzo-*p*-dioxin (TCDD) on the development and function of the EPB.

Methods: Timed-pregnant C57BL/6J mice were gavaged with corn oil or TCDD (10 μg/kg body weight) on gestation day 12. Embryos were harvested on embryonic day (E) 15, E16, E17, and postnatal day (PND) 1.

Results: A skin permeability assay showed that TCDD accelerated the development of the EPB, beginning at E15. This was accompanied by a significant decrease in transepidermal water loss (TEWL), enhanced stratification, and formation of the stratum corneum (SC). The levels of several ceramides were significantly increased at E15 and E16. PND1 histology revealed TCDD-induced acanthosis and epidermal hyperkeratosis. This was accompanied by disrupted epidermal tight junction (TJ) function, with increased dye leakage at the terminal claudin-1–staining TJs of the stratum granulosum. Because the animals did not have enhanced rates of TEWL, a commonly observed phenotype in animals with TJ defects, we performed tape-stripping. Removal of most of the SC resulted in a significant increase in TEWL in TCDD-exposed PND1 pups compared with their control group.

Conclusions: These findings demonstrate that *in utero* exposure to TCDD accelerates the formation of an abnormal EPB with leaky TJs, warranting further study of environmental exposures, epithelial TJ integrity, and atopic disease.

Citation: Muenyi CS, Leon Carrion S, Jones LA, Kennedy LH, Slominski AT, Sutter CH, Sutter TR. 2014. Effects of *in utero* exposure of C57BL/6J mice to 2,3,7,8-tetrachlorodibenzo-*p*-dioxin on epidermal permeability barrier development and function. Environ Health Perspect 122:1052–1058; http://dx.doi.org/10.1289/ehp.1308045

## Introduction

Formation of a competent epidermal permeability barrier (EPB) is essential to terrestrial life. This barrier prevents desiccation and protects the body against microbes, as well as physical and chemical insults. The EPB is established *in utero* during embryonic development and maintained throughout life. In humans, EPB formation occurs between 20 and 24 weeks of gestation (Hardman et al. 1999). Barrier formation in mice starts at embryonic day (E) 16 and is completed by E17.5 (Hardman et al. 1998). The stratum corneum (SC), the outermost layer of the epidermis, contributes greatly to the functioning of the EPB. The SC is made up of corneocytes: terminally differentiated keratinocytes, highly cross-linked by transglutaminases with cornified envelope proteins such as loricrin, involucrin, filaggrin, and small proline-rich proteins. These anucleated cells are embedded in a lipid matrix of ceramides, cholesterol, and free fatty acids to form the “brick and mortar” structure of the SC that seals the epidermis and provides protection to the skin (Menon et al. 2012).

In addition to the SC, tight junctions (TJs) provide additional barrier function to the skin. TJs are distributed in the stratum granulosum (SG) layer, located beneath the SC. TJs seal the intercellular spaces between cells and regulate paracellular transport of water, ions, and solutes (Proksch et al. 2008). Several studies have demonstrated that the EPB is compromised in mice that have disrupted TJs (Furuse et al. 2002; Turksen and Troy 2002). A defective EPB (Boguniewicz and Leung 2011; De Benedetto et al. 2012; Proksch et al. 2008) and decreased expression of TJ proteins have been reported in patients with atopic dermatitis (De Benedetto et al. 2011) and psoriasis (Kirschner et al. 2010). A claudin-1 deficiency has been associated with NISCH syndrome (neonatal ichthyosis-sclerosing cholangitis, a familial form of ichthyosis) (Morita et al. 2011). Disruption of epithelial TJ function also has been reported in the lungs of patients with asthma (Xiao et al. 2011) and in the intestines of people with inflammatory bowel diseases (Schulzke et al. 2009).

2,3,7,8-Tetrachlorodibenzo-*p*-dioxin (TCDD) is a ubiquitous environmental pollutant and the most potent aryl hydrocarbon receptor (AHR) ligand. The hallmark of TCDD toxicity in humans is chloracne (Panteleyev and Bickers 2006), characterized by epidermal acanthosis and hyperkeratosis, and hyperkeratinization and metaplasia of the sebaceous glands, with comedone formation. In cultures of normal human epidermal keratinocytes, treatment with TCDD increased the expression of many genes involved in cornification and EPB formation (Kennedy et al. 2013; Sutter et al. 2009, 2011), leading to enhanced rates of terminal differentiation (Sutter et al. 2009) and ceramide biosynthesis (Kennedy et al. 2013). In organotypic culture of a normal human keratinocyte cell line, TCDD has been reported to cause early onset of terminal differentiation, and premature and irregular expression of filaggrin and involucrin, with marked thickening of the keratinized cell layers and hyperkeratosis (Loertscher et al. 2001).

In haired rodents, chloracne-like skin lesions are usually absent after TCDD treatment; although in one study of B6C3F1 mice, such lesions were observed after 2 years of treatment with the dioxin-like compound 3,3´,4,4´-tetrachlorazobenzene (Ramot et al. 2009). Compared with haired mice, hairless mouse strains (*hr/hr* mutants) are very sensitive to TCDD-induced lesions characteristic of chloracne, including epidermal hyperplasia and hyperkeratinization and involution of the sebaceous glands (Puhvel and Sakamoto 1988). Studies of *in utero* exposure of C57BL/6J embryos to TCDD by gavage of the dam showed accelerated expression of filaggrin at E16 and the presence of a morphologically well-organized epidermis in these TCDD-exposed embryos (Loertscher et al. 2002). In a subsequent study, we reported that *in utero*–exposed C57BL/6J mouse embryos exhibited accelerated formation of the EPB by 1 day, and that in normal human keratinocytes, many of the genes of the epidermal differentiation complex responded to TCDD (Sutter et al. 2011). Of particular interest, filaggrin gene expression was shown to be directly regulated by AHR-binding to the filaggrin xenobiotic response element in response to TCDD (Sutter et al. 2011). Because of the emerging association between disrupted EPB function and inflammatory diseases of the skin, we performed studies to determine whether the acceleration of the EPB by *in utero* exposure to TCDD resulted in normal or abnormal structure and function of the EPB.

## Materials and Methods

*Animals*. We purchased time-mated, presumed-pregnant C57BL/6J mice from Jackson Laboratory (Bar Harbor, ME), defining E1 as the day after a vaginal plug was observed. We housed two to five dams in clear plastic cages and maintained a 12:12-hr light:dark cycle in a temperature-controlled room (24°C ± 1°C) with 35% ± 4% relative humidity, providing food and water to the mice *ad libitum*. Pregnant dams were euthanized by asphyxiation with carbon dioxide, and the entire uterus with embryos was removed. We dissected the embryos from the embryonic sacs and rinsed them twice in ice-cold (4°C) phosphate buffered saline (PBS), pH 7.4. Postnatal day (PND) 1 pups were euthanized by intraperitoneal injection of SOMNASOL^TM^ Euthanasia-III solution (1 mL/4.5 kg body weight; National Drug Code no. 11695-4829-1; Butler Schein Animal Health, Dublin, OH). Animal research protocols were approved by the University of Memphis Institutional Animal Care and Use Committee; animals were treated humanely and with regard for alleviation of suffering.

*Experimental design*. We fed dams Teklad Global 18% Protein Rodent Diet 2018 until E9 and then fed Teklad Global 16% Protein Rodent Diet 2016 (both from Harlan Teklad, Madison, WI). On E12, we weighed the dams and randomly distributed them into eight groups (corn oil and TCDD groups at E15, E16, E17, and PND1); dams were treated by oral gavage with corn oil or a single dose of 10 μg TCDD in 110 μL corn oil/kg body weight. We harvested the embryos at E15, E16, and E17 and pups on PND1.

*Transepidermal water loss (TEWL)*. We harvested embryos and PND1 pups and rinsed them twice in PBS, allowed them to air dry for 5 min, and measured TEWL (in grams per square meter per hour) at the dorsal posterior region using the Delfin VapoMeter with a 4.5-mm nail adapter attached (Delfin Technologies Ltd, Stamford, CT).

*Skin permeability assay*. We performed an EPB assay using the β-galactosidase substrate 5-bromo-4-chloro-3-indolyl β-d-galactopyranoside (X-gal) according to a published method (Hardman et al. 1998), as previously described (Sutter et al. 2011). Briefly, the embryos and pups were incubated in the X-gal reaction mixture for 24 hr at room temperature, then fixed in 4% paraformaldehyde at 4°C for 24 hr, and subsequently transferred to 70% alcohol. Digital images were quantified as described previously (Sutter et al. 2011).

*Histology*. We fixed whole embryos or PND1 mice for 24 hr at 4°C in 4% paraformaldehyde, pH 7.4, followed by 20% sucrose for 24 hr at 4°C. We embedded the fixed animals in optimal cutting temperature (OCT) medium (Tissue-Tek; Sakura Finetek USA, Torrance, CA) and prepared a sagittal section of 10-μm thickness using a microtome cryostat. We stained the sections with hematoxylin and eosin (H&E) reagents and visualized the sections using a Nikon Eclipse E800 microscope (Nikon, Melville, NY). For toluidine blue staining, an approximately 5-mm piece of dorsal skin was fixed in 2.5% glutaraldehyde plus 2.5% paraformaldehyde in 0.1 M sodium cacodylate buffer (pH 7.4) at 4°C. The samples were post-fixed in 2% osmium in 0.1 M sodium cacodylate buffer (pH 7.4), embedded in Epon 812 (Polysciences, Warrington, PA), and cut in semithin sections (800 nm) on an Ultracut E microtome (Reichert Technologies, Depew, NY). We applied filtered toluidine blue staining solution (0.5% toluidine blue plus 1% borax in deionized water) to dried semithin sections on a 60°C hot plate and stained them for 2 min, then rinsed the stained slides under running tap water. We cleared the sections by dipping them into a 95% acid-alcohol solution (50 mL of 95% ethanol plus one drop of glacial acetic acid solution). We subsequently rinsed the slides under running tap water to remove excess acid alcohol and let the slides dry before mounting with Cytoseal-XYL mounting medium (Richard-Allan Scientific, Kalamazoo, MI). We evaluated the slides using a Nikon Eclipse E800 microscope.

*Ultrathin section transmission electron microscopy (TEM)*. We cut tissue sections (50–70 nm) using an Ultra Cut UCT (Leica Mikrosysteme GmbH, Vienna, Austria) with a Diatome diamond knife (Electron Microscopy Sciences, Hattfield, PA). We mounted the cut sections on Formvar-carbon–supported copper grids and air dried them in a clean, covered area. We stained the tissue sections with aqueous 4% uranyl acetate for 30 min at room temperature and rinsed them with deionized water. The moist grids were stained for 2 min with Reynold’s Lead Citrate (Electron Microscopy Sciences), rinsed, and allowed to dry completely in a clean, covered area. We analyzed the dried grids with a Jeol 1200EX II TEM (Jeol USA Inc., Peabody, MA) using 60 KV or 80 KV.

*Epidermal lipid analysis*. We weighed whole embryos or PND1 pups and extracted the epidermal lipids in chloroform:methanol (1:2 vol/vol) by vortexing at moderate speed for 2 min. The organic phase was dried under liquid nitrogen, redissolved in chloroform:methanol (1:1), and analyzed by high-performance thin-layer chromatography (HPTLC) as previously described (Tran et al. 2012).

*TJ permeability assay*. We analyzed TJ function according to a published method (Furuse et al. 2002). We injected 50 μL of a 10-mg/mL biotin-SH (EZ-Link Sulfo-NHS-LC-Biotin; catalog no. 21335; ThermoScientific, Pittsburgh, PA) solution in PBS, pH 7.4, containing 1 mM calcium chloride into the dermis on the backs of PND1 pups. After a 30-min incubation at room temperature, we cryopreserved the whole pups in OCT medium, and cryosectioned sagittal sections (10 μm). We fixed the tissue sections in 95% ethanol at 4°C for 30 min, followed by 100% acetone at room temperature for 1 min. We incubated the tissues in 10% normal goat serum blocking solution for 30 min at room temperature, subsequently incubated them in claudin-1 monoclonal antibody (1:200; catalog no. 51-9000; Invitrogen, Grand Island, NY) for 30 min, and washed them three times with PBS for 10 min each wash. The tissue sections were subsequently incubated in a solution of Alexa Fluor® 488 Goat Anti-Rabbit IgG (H+L) (1:2,000; catalog no. A11008; Invitrogen) and Streptavidin, Alexa Fluor® 568 conjugate (1:200; catalog no. S-112260; Invitrogen) for 30 min, washed with PBS three times for 10 min each, mounted with ProLong® Gold Antifade Reagent with DAPI (catalog no. P-36931; Invitrogen) and let cure overnight. We visualized the sections using a Nikon A1 laser-scanning confocal microscope. We counted claudin-1–positive sites of the SG, with or without stops, for the diffusion of biotin-SH toward the skin surface. We counted at least three visual fields per sample, and analyzed a total of three pups from three different dams per treatment condition.

*SC tape stripping*. We euthanized PND1 pups as described above and measured TEWL after sequential tape stripping of the SC layers of the dorsal posterior skin using adhesive tape (catalog no. 159015R; 19 mm × 13 mm; ThermoScientific). We performed six tape strippings to remove most of the SC (Tsai et al. 1991).

*Statistical analysis*. We expressed the data as means ± SDs. We compared age-matched control and TCDD-exposed groups using Student’s *t*-test; a level of *p* < 0.05 was set as statistically significant for all comparisons.

## Results

**Figure 1:**
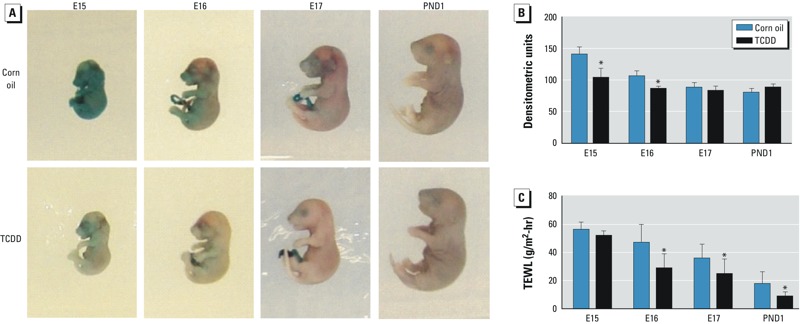
Accelerated EPB formation and function in developing murine skin of TCDD-exposed embryos. (*A*) Representative photographs taken after the X-gal skin permeability assay. (*B*) Quantification of photographs from the X-gal skin permeability assay. (*C*) TEWL measured at the dorsal posterior region of murine skin. Data are means ± SDs (*n *≥ 6). *p < 0.05, compared with age-matched corn oil control samples by Student’s *t*-test.

*TCDD accelerates EPB formation and function*. Previously, we reported that 3-day *in utero* exposure to TCDD accelerated EPB formation in C57BL/6J mice by 1 day, beginning at E15 (Sutter et al. 2011). In the present study, embryos were continuously exposed *in utero* to TCDD beginning on E12. Development of the EPB, measured as exclusion of an X-gal substrate of endogenous epidermal β-galactosidase, was accelerated by 1 day in TCDD-exposed embryos, beginning at E15 and continuing to E16. By E17 and continuing to PND1, we observed no differences between the control and TCDD-exposed animals, with complete development of the EPB by PND1 (Figure 1A,B). To evaluate the integrity of EPB function, we measured TEWL in the dorsal posterior region of the embryos and PND1 pups. TEWL readings were significantly lower in the TCDD-exposed mice at E16, E17, and PND1 compared with their age-matched corn-oil controls (Figure 1C), indicating that *in utero* exposure to TCDD significantly accelerated the function of the EPB.

**Figure 2:**
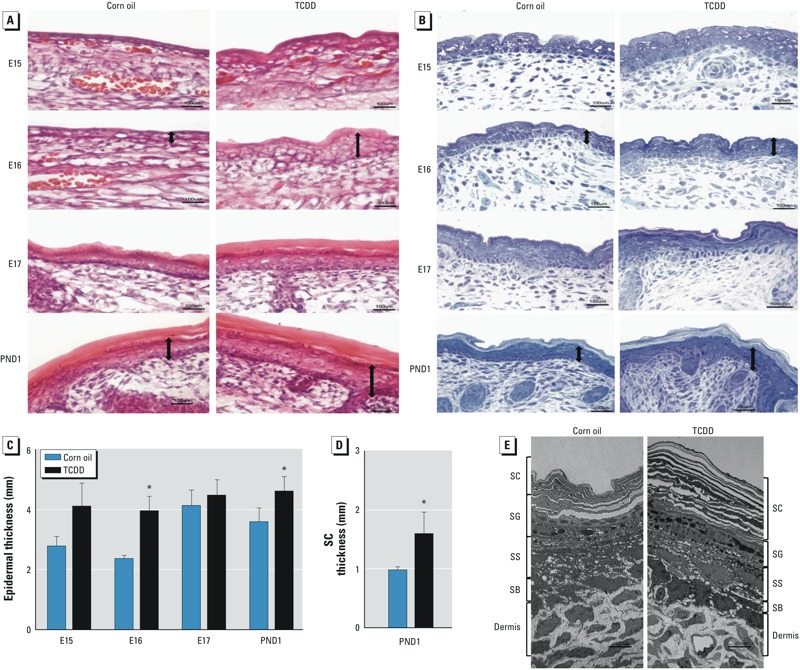
Epidermal abnormalities after *in utero* TCDD exposure. (*A*) Photomicrograph of epidermis showing H&E staining of frozen sections of representative embryos and pups. (*B*) Photomicrographs of semithin-sectioned murine skin stained with toluidine blue. Images are representative for each time point and treatment condition. Double-headed arrows identify examples of the relative thickness of the epidermal layers corresponding to acanthosis. Bars = 10 μm. (*C*) Quantification of epidermal thickness by microscopy. (*D*) Quantification of SC thickness at PND1 by microscopy. In *C* and *D*, data are means ± SDs (*n* = 3). (*E*) Photomicrograph of ultrathin skin section of PND1 mice analyzed by TEM. Bar = 600 μm. Abbreviations: SB, stratum basale; SS, stratum spinosum. *p < 0.05, compared with age-matched control samples by Student’s *t*-test.

*TCDD exposure results in epidermal acanthosis and hyperkeratosis*. Topical application of TCDD on hairless mice skin has been reported to cause epidermal hyperplasia (Puhvel and Sakamoto 1988). However, Loertscher et al. (2002) previously reported that *in utero* exposure of C57BL/6J embryos to TCDD did not alter the histology of the skin. Contrary to Loertscher et al. (2002), histology with H&E and toluidine blue staining indicated that TCDD was associated with an early onset of epidermal hyperplasia beginning at E15 (Figure 2A,B). Significant thickening of the epidermis was observed at E16 and PND1 (Figure 2A,B, double-headed arrows; Figure 2C), indicating epidermal acanthosis in response to exposure to TCDD. Similarly, the SC was readily apparent in TCDD-exposed embryos as early as E16 (Figure 2A,B), and measurement of the SC at PND1 revealed that this layer was about twice as thick in the TCDD-exposed pups compared with their corn-oil controls (Figure 2D). The observed thickening of the SC in TCDD-exposed mice is indicative of a pronounced epidermal hyperkeratosis. Enhanced thickening of the epidermis and SC by TCDD was confirmed using ultrathin-section TEM (Figure 2E). Significant thickening of the SC was not due to an increase in the number of SC layers because the number of SC layers—approximately 10–12 layers—was similar in the corn-oil control and TCDD-exposed mice.

**Figure 3:**
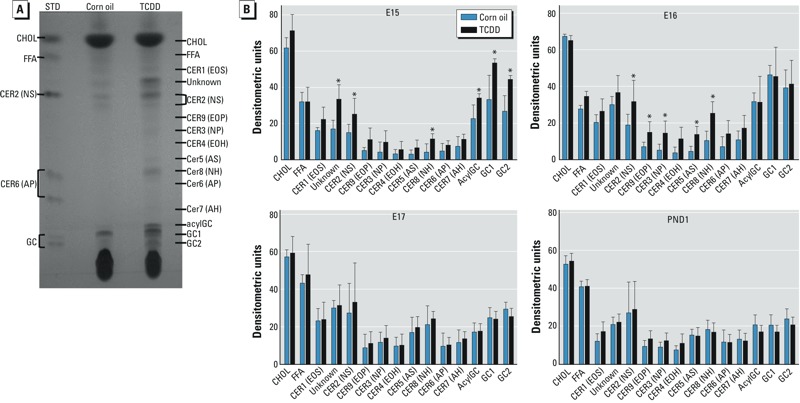
Increases in the level of certain ceramides at E15 and E16 in TCDD-exposed embryos. (*A*) Representative photograph of epidermal lipids separated by HPTLC; the ceramide structures are named based on the sphingoid base (S, sphingosine; P, phytosphingosine; H, 6-hydroxysphingosine) and the *N*-acyl fatty acid (A, α-hydroxy group; O, ω-hydroxy group; E, acylated in the ω-hydroxy position). Abbreviations: acylGC, acylglucosylceramide; CER, ceramide; CHOL, cholesterol; FFA, free fatty acid; GC, glucosylceramide; STD, standard. (*B*) Quantification of lipid bands by densitometry. Data are means ± SDs (*n *≥ 3). *p < 0.05, compared with age-matched control samples by Student’s *t*-test.

*TCDD increases epidermal ceramide levels*. The lipid-enriched matrix of the SC is composed of cholesterol, free fatty acids, and ceramides. Ceramides are the predominant lipids in the SC (Uchida and Holleran 2008). In cultures of normal human epidermal keratinocytes, we have observed increases in several classes of ceramides, without changes in cholesterol or free fatty acids (Kennedy et al. 2013). Here, we investigated whether TCDD altered the composition of lipids in developing murine skin. We extracted epidermal lipids and separated them by HPTLC (Figure 3A), with assignments based on standards and our previous analyses (Kennedy et al. 2013; Tran et al. 2012). The levels of short-chain ceramides (NS and NH) and the ceramide precursors acylglucosylceramide (acylGC) and glucosylceramide (GC) were increased in TCDD-exposed embryos at E15 (Figure 3B). At E16, levels of acylGC and GC were similar in the control and TCDD-exposed mice. However, additional short-chain ceramides (NS, NP, AS, and NH) and the long-chain ceramide (EOP) were elevated in the TCDD-exposed embryos (Figure 3B). At E17 and PND1, ceramide levels were similar in the control and TCDD samples. Given that ceramides are important components of the SC, the observed elevation of ceramides at E15 and E16 might be a contributing factor to the accelerated barrier formation and function observed in the *in utero* TCDD-exposed mice (Figure 1). The levels of cholesterol and free fatty acids were unaffected by TCDD, consistent with what we previously observed in human keratinocytes (Kennedy et al. 2013).

**Figure 4:**
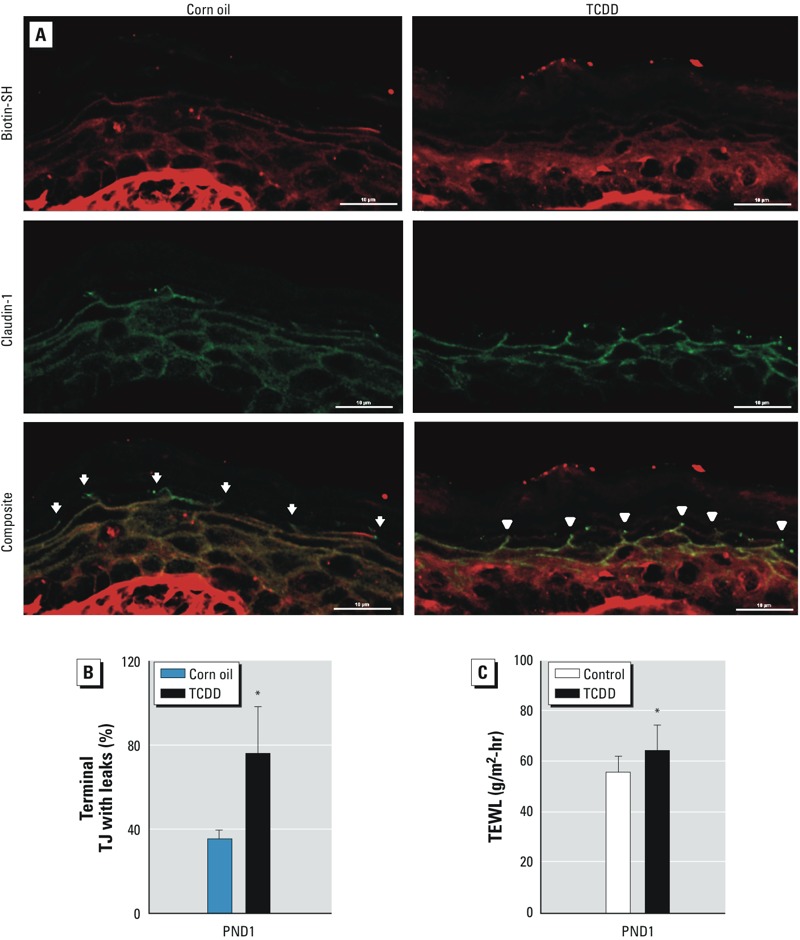
Disruption of TJ permeability barrier after *in utero* TCDD exposure. (*A*) Photomicrograph of PND1 murine skin exposed to corn oil or TCDD. Arrows indicate claudin-1–positive sites with biotin-SH stops, and arrowheads indicate claudin-1–positive sites without biotin-SH stop. Bars = 10 μm. (*B*) Quantification of claudin-positive sites for terminal TJs without biotin-SH stops (≥ 3 visual fields per sample were counted, and 3 pups per treatment condition were analyzed). A total of 87 terminal TJs were counted in corn-oil samples; 30 of these terminal TJs had leaks for biotin-SH. In TCDD-exposed pups, 104 terminal TJs were counted, and 72 of these terminal TJs had leaks for biotin-SH. (*C*) TEWL after SC removal in PND1 mice. The dorsal skin of mice was tape stripped six times to remove most of the SC before TEWL was measured. At least 22 pups from 4 dams were assayed in the control or TCDD-exposed group. Values are means ± SDs. *p < 0.05, compared with age-matched control samples by Student’s *t*-test.

In utero *exposure to TCDD disrupts TJ function in PND1 pups*. In addition to the SC, TJs of the lateral membrane of the SG contribute to the paracellular water-and-ion barrier that is essential to EPB function (Furuse et al. 2002). Because of the importance of these TJs to the EPB, we investigated whether *in utero* exposure to TCDD altered this function. We injected biotin-SH dye into the dermis of PND1 mice and monitored the diffusion of this dye from the dermis through the epidermis, quantifying whether the biotin-SH dye crossed or stopped at the claudin-1–staining TJs located in the apical region of the SG. We examined terminal TJs for biotin-SH stop or leakage sites by co-localization (Figure 4A, composite) of claudin-1 (Figure 4A, green) and biotin-SH dye (Figure 4A, red). In the control pups, biotin-SH dye stopped at > 60% of the claudin-1–staining TJs. In the TCDD-exposed pups, nearly 80% of these TJs had leaks for biotin-SH (Figure 4B), indicating that TCDD is disrupting the TJ barrier function. To understand why TCDD-exposed mice with disrupted TJ barrier showed significantly lower TEWL (Figure 1C)—which is contrary to what was expected—we investigated the idea that the observed hyperkeratosis in the TCDD-exposed mice compensated for the leaky TJs, thus diminishing water loss in this abnormal EPB. In order to test this hypothesis, we performed six sequential tape strippings of the dorsal posterior skin of control and TCDD-exposed PND1 mice to remove most of the SC. Our data indicate that the removal of the SC resulted in a significant increase in TEWL in the TCDD-exposed mice compared with their age-matched tape-stripped controls (Figure 4C). This result is consistent with the idea that the thick SC layer in TCDD-exposed murine skin acts to prevent water loss, even in the presence of a disrupted TJ barrier.

## Discussion

The epidermis serves as the first line of defense and protection against environmental pathogens, allergens, and toxins, as well as preventing the loss of water and ions. In mice, formation of the EPB begins at E16 and is completed by E17.5. We previously reported that TCDD accelerated the timing of EPB formation in C57BL/6J mice starting at E15 (Sutter et al. 2011). Here, we confirm our previously published data and also report that, at E17, barrier formation is completed in C57B/6J embryos exposed *in utero* to TCDD or corn oil.

The SC, with its corneocytes and lipid matrix and the TJs of the SG constitute the EPB function of the epidermis. Ceramides are the major lipid component in the lipid matrix of the SC. Microarray and lipid analyses previously published by our laboratory showed that the expression of approximately 75% of genes involved in *de novo* ceramide biosynthesis, as well as the levels of eight classes of ceramides, were increased in TCDD-exposed human keratinocytes (Kennedy et al. 2013). In the present developmental animal study, we found that the accelerated barrier formation by TCDD at E15 is associated with an increased accumulation of the ceramide precursors acylGC and GC as well as the short-chain ceramides NS and NH. At E16, we observed elevated levels of short-chain (NS, NP, AS, and NH) and long-chain (EOP) ceramides in TCDD-exposed embryos, which corresponded with the accelerated formation and function of the EPB to exclude X-gal. However, elevated levels of ceramides at E15 and E16 were not accompanied by increases in cholesterol or free fatty acids. This imbalanced ratio of ceramide to free fatty acids and cholesterol might affect lamellar body formation and alter barrier homeostasis. Such alterations of skin lipids have been implicated in skin disorders such as lamellar ichthyosis (Feingold 2007; Schmuth et al. 2001; Uchida and Holleran 2008).

Exposure to TCDD has been reported to cause chloracne in humans (Panteleyev and Bickers 2006), and limited animal studies have reported that topical application of TCDD on hairless mice resulted in thickening of the epidermis (acanthosis) and SC (hyperkeratosis) (Panteleyev et al. 1997; Puhvel and Sakamoto 1988). In one study, Loertscher et al. (2002) reported that *in utero* TCDD exposure did not alter the normal epidermal morphogenesis even though they observed premature expression of filaggrin at E16. However, in the present study, we found that *in utero* TCDD exposure altered the histology of the epidermis, resulting in epidermal hyperplasia beginning at E15. Thickening of the epidermis was observed in the TCDD-exposed embryos and PND1 pups. In addition, significant epidermal hyperkeratosis was observed at PND1. Our data clearly indicate abnormal, but accelerated, EPB formation after *in utero* TCDD exposure. Epidermal acanthosis and hyperkeratosis are histopathological characteristics that are commonly observed in chloracne (Panteleyev and Bickers 2006) and epidermolytic hyperkeratosis, a genetic disorder associated with keratin mutations (Müller et al. 2006; Reichelt et al. 1999). The SC and TJs provide the physical barrier components of the epidermis. Disruption of the integrity of the SC or TJ barrier impairs the normal functioning of the EPB. Using a biotin-SH TJ assay we showed that TCDD can disrupt the TJ barrier in PND1 pups, resulting in the leakage of the dye across the TJs. Several animal studies have reported that mice with defective TJs have compromised EPB function and show enhanced TEWL (Furuse et al. 2002; Sugawara et al. 2013; Tunggal et al. 2005; Turksen and Troy 2002). Contrary to these reports, in the present study we observed that TCDD-exposed embryos showed unexpectedly lower rates of TEWL from E16 to PND1, suggesting that there might be a compensatory mechanism preventing excessive water loss via the defective TJ barrier. Kuramoto et al. (2002) previously reported that grafted mature transglutaminase 1 (TGase1)–deficient murine skin with remarkable epidermal hyperplasia and hyperkeratosis showed lower TEWL, similar to control TGase1-proficient mice. However, removal of the thick epidermal hyperkeratosis resulted in an increase in TEWL (Kuramoto et al. 2002). In a similar light, we report here that removal of the SC by tape stripping resulted in a significant increase in TEWL in the TCDD-exposed pups compared with the corn-oil control pups. Thus, the observed hyperkeratosis in TCDD-exposed pups may act to compensate for the disrupted TJ component of the EPB.

In addition to the SC, TJs contribute to the paracellular water-and-ion barrier that is present in the SG. Disruption of the TJ barrier has been linked to atopic diseases in humans (Boguniewicz and Leung 2011; De Benedetto et al. 2012), and a few studies of TCDD have shown that exposure to this environmental pollutant exacerbates atopic diseases in an animal model (Ito et al. 2008) and in humans (Kim et al. 2003; Kimata 2003). Mice expressing a keratin 14–driven constitutively active AHR exhibit skin lesions with itching that are consistent with atopic dermatitis (Tauchi et al. 2005), and one study of Korean Vietnam veterans reported a statistically significant association between the incidence of eczema and Agent Orange exposure (Kim et al. 2003). Nonetheless, the role of TCDD in the causation of atopic dermatitis remains controversial. Whereas some studies have found that TCDD exacerbates atopic dermatitis in NC/Nga mice (Ito et al. 2008), increases IgE production in B cells from patients with atopic diseases (Kimata 2003), and disrupts mucosal immunity in the gut and sensitizes C57BL/6J mice to oral allergens (Kinoshita et al. 2006), other studies have reported that exposure to TCDD suppressed allergic immune response to ovalbumin, dust, and peanuts in laboratory animals (Luebke et al. 2001; Schulz et al. 2011; Tarkowski et al. 2010) and failed to induce atopic dermatitis in NC/Nga mice (Fujimaki et al. 2002). Of interest, all of these studies of TCDD and atopic disease have focused on the immunological responses occurring after TCDD exposure. Although the immune component of atopic disease should not be understated, the emerging understanding of the role of a defective EPB as an underlying cause of several atopic diseases (Boguniewicz and Leung 2011; De Benedetto et al. 2012; Proksch et al. 2008) indicates the need for further study of this important aspect of biology. For example, it is now understood that a compromised EPB is required for allergens to enter the epidermis and elicit inflammatory or hypersensitive reactions (Boguniewicz and Leung 2011; De Benedetto et al. 2012). In addition, loss-of-function mutations in the filaggrin gene have been identified as a major predisposing factor for atopic dermatitis (Palmer et al. 2006).

Also of interest, albeit from a therapeutic perspective, van den Bogaard et al. (2013) recently reported that activation of the AHR by coal tar in a submerged culture of human keratinocytes and human organotypic skin from patients with atopic dermatitis enhanced epidermal differentiation and thickening of the SC in the skin equivalents; in biopsies from patients treated with coal tar, the expression of filaggrin and other markers of differentiation were increased. Whether these potentially beneficial effects of coal tar will be limited to atopic dermatitis associated with filaggrin mutations or whether AHR activation may provide general benefit to this inflammatory skin disease is currently unknown. Similarly, whether the difference in perspective (i.e., therapeutic vs. toxic) between van den Bogaard et al. (2013) and the work presented here represents differences between adult and perinatal exposure, differences between mice and humans, or differences between long-acting ligands such as TCDD and shorter-acting, metabolized AHR agonists, such as polycyclic aromatic hydrocarbons, remains unknown. Finally, the effects reported by van den Bogaard (2013) did not consider additional aspects of the EPB such at the TJ and lipid components. Nonetheless, because of the importance of both developmental susceptibility and the need for mechanism-based treatments for inflammatory skin disease, all of these questions and their answers require further elaboration.

## Conclusions

We found that the timing of the formation of an abnormal EPB was accelerated after *in utero* exposure to TCDD. The histopathology of this abnormal barrier was characterized by acanthosis and hyperkeratosis. Moreover, TCDD disrupted the TJ function of the epidermis. Tape stripping of control and TCDD-exposed mice indicated that epidermal hyperkeratosis compensated for excessive TEWL from the disrupted barrier. These results indicate that TCDD has the potential to *a*) induce or exacerbate cutaneous skin diseases by disrupting EPB integrity and function, and *b*) identify the developing epidermis as a target for *in utero* exposure to this ubiquitous environmental pollutant.
